# Validation of the professional self-identity questionnaire for medical students during clinical practice in Japan

**DOI:** 10.5116/ijme.610d.104b

**Published:** 2021-08-31

**Authors:** Junji Haruta, Takami Maeno, Ayumi Takayashiki, Ryohei Goto, Sachiko Ozone, Tetsuhiro Maeno

**Affiliations:** 1 Medical Education Center, School of Medicine, Keio University, Japan; 2 Department of Primary Care and Medical Education, Faculty of Medicine, University of Tsukuba, Japan; 3 Department of General Medicine and Primary Care, Faculty of Medicine, University of Tsukuba, Japan

**Keywords:** Professional identity, community-based medical education, curriculum evaluation, clinical-clerkship, cultural responsiveness, Japan

## Abstract

**Objectives:**

To validate the Professional Self Identity Questionnaire (PSIQ) for medical students during clinical practice.

**Methods:**

We conducted a single-year longitudinal questionnaire study using the PSIQ. The PSIQ rates the nine items of "teamwork", "communication", "conducting assessment", "cultural awareness", "ethical awareness", "using records", "dealing with emergencies", "reflection", and "teaching" on a scale of 1-7 points. The study participants consisted of 118 fifth- and sixth-grade medical students who completed a mandatory 4-week clinical practice in a community-based medical education (CBME) curriculum. The data were collected before and after the CBME curriculum and after clinical practice at the time of graduation. To validate the internal structure of the PSIQ, we calculated Cronbach's alpha in the three phases. Additionally, to assess construct validity, we analyzed the trends and differences in each of the nine items of the PSIQ using repeated measures analysis of variance (ANOVA). We also showed the differences in effect size before and after the CBME curriculum.

**Results:**

The data of 105 medical students were analyzed. Cronbach's alpha in the three phases was 0.932, 0.936, and 0.939, respectively. PSIQ scores increased progressively for all items, and the F-test for repeated measures ANOVA of nine items' average score across the three phases showed a significant difference F_(2,208)_ = 63.59, p<0.001. The effect size for professional identity of cultural awareness before and after the CBME curriculum was 0.67, or medium.

**Conclusions:**

We validated the PSIQ for medical students during clinical practice. Reflecting on professional identity may provide an opportunity for meaningful feedback on readiness to become a doctor.

## Introduction

Professional identity is a 'state of mind' – namely, the identification of oneself as a member of a professional group.^1^ It is defined as "the attitudes, values, knowledge, beliefs and skills shared with others within a professional group".^2^ The development of professional identity has been described as an ongoing process which is influenced by several factors, including clinical experience and professional socialization.^3^ In the last two decades, in accompaniment with advances in medical genetics and other technologies, healthcare providers have increasingly had to deal with complex ethical issues in which medical knowledge alone is not enough; examples include newly arising infectious diseases, socioeconomic problems and the aging population.^4^ In this context, the need for professionalism by individual physicians is growing. In medical education, the aim of professionalism education has expanded to include the development of professional identity.^5^

In undergraduate medical education, professional identity is influenced by various aspects of the university curriculum.^6^^-^^8^ The development of professional identity plays an important role in promoting students' sense of responsibility and in defining themselves within their professional role.^9^ In particular, the professional identity of medical students during clinical practice changes dynamically as their identities are constructed and deconstructed through multiple departmental rotations.^10^ For medical students, professional identity is impacted by any and all of experiential learning, early clinical experience, role models, dialogues, socialization, and relationships between physicians and other healthcare professions.^7^^,^^11^^,^^12^ Students' behavior as healthcare providers is also influenced by the opportunity to engage in meaningful interaction with and provide constructive feedback to multiple healthcare professionals.^7^

Within the clinical practice, the community-based medical education (CBME) curriculum creates the opportunity to formulate such a professional identity.^13^ One important reason for this is that the students typically find themselves in smaller groups in their CBME rotation than in other clinical practice rotations, which provides greater opportunity to both converse with and develop close relationships with supervising physicians and multi-healthcare professionals, and to learn cross-cultural community needs.^14^ In CBME, medical students experience different aspects of professional competencies, such as cultural awareness.^15^ Through these experiences, students encounter patients and issues in the context of their lives and localities and benefit from experiential learning through their involvement in patient care.^16^^-^^18^To date, however, few validated questionnaires have been developed to assess professional identity formation during clinical practice, including CBME.^15^

In this study, we focused on the Professional Self Identity Questionnaire (PSIQ). Originally developed to measure professional identity in the healthcare professions,^1^ the PSIQ has been used for medical and other students and can also be used to evaluate professional identity in multiple professions. It assesses nine professional identity items in a multifaceted manner and is easy to use at any level.^19^^,^^20^ Here, we aimed to validate the PSIQ to measure professional identity formation in medical students during clinical practice, including CBME.

## Methods

### Study design and participants

This longitudinal questionnaire study was conducted to test the validity of the PSIQ. The study participants consisted of 118 fifth- and sixth-grade medical students who completed a mandatory 4-week clinical practice in the CBME curriculum between September 2018 and May 2019. We explained to the students that this survey would be conducted as program evaluation and research and that their grades would not be affected if they did not agree to participate. This study was approved by the Ethics Committee of the Faculty of Medicine, the University of Tsukuba (No. 1329).

### Data collection method

The PSIQ includes nine inventory items, namely "teamwork", "communication", "conducting assessment", "cultural awareness", "ethical awareness", "using records", "dealing with emergencies", "reflection", and "teaching".^1^ Each item is rated on a 7-point Likert scale. Through engagement with each of its nine items of professional activity, this instrument measures the sense of students in identifying their current position on a continuum between 'first-day student' and 'qualified doctor'. For this research, we set 1 point as "equivalent to the first day of clinical practice" and 7 points as "equivalent to the first day of initial residency". The original study was validated in a sample of 496 medical students across multiple phases of education and had a reported overall internal reliability (Cronbach's alpha) of 0.93.^1^ In our present study, two researchers (JH and SO) independently translated the English language version of the PSIQ into Japanese. Three other authors (TM, AH, TM) then finalized the content and comprehensibility to produce a Japanese version.

### Setting

First, we describe aspects of medical education in Japan, which in context may have influenced our findings.^21^^,^^22^ In Japan, eligibility to enter medical school is assessed in high school graduates. The standard undergraduate medical education program is six years. Typically, the initial phase of undergraduate medical education contains, to varying degrees, general education in subjects such as biology, chemistry, physics, and mathematics, as well as a wide range of liberal arts subjects. In parallel with the liberal arts, medical students study basic medicine for 1-2 years, followed by pre-clinical education for 1-2 years. Medical students must pass the CBT (Computer-based testing) and Pre-OSCE (Objective Structured Clinical Examination) exams prior to clinical practice to earn the title "Student doctor". After this, from the fourth or fifth grade of medical school, they spend one to one and a half years in a university hospital or community hospital or clinic in a community in small groups of 4-5 students. After a two-year clinical practice, they must pass their University's graduation exam and the post-clinical-clerkship OSCE to qualify for graduation, and then pass the annual national medical examination. The academic year starts on April 1 and ends on March 31.

Second, we focused on clinical practice at the University in this study. Clinical practice at the University is implemented from October in Year 4 to June in Year 6, for a total of 78 weeks, which made it the second-longest among Japanese medical schools at that time. This clerkship program is a part of the mandatory 4-week CBME (Community-based Medical Education) curriculum, which is conducted from October in the second half of Year 5 to May in Year 6. In clinical practice, the 4-week CBME curriculum is an essential component of education for medical professionals at the University. The 6-year pre-graduate curriculum provides students with the essential foundational competence required of all medical professionals, including medical ethics, primary care, health promotion, professionalism, and interprofessional collaboration. The CBME curriculum is the clinical practice component of this essential knowledge for medical professionals.

The CBME curriculum aims to help students: 1) understand the expertise of family physicians, who provide appropriate medical care in various clinical settings; 2) understand the health issues of citizens, patients, and families from the perspective of the local healthcare system; and 3) acquire clinical reasoning skills.

Every four weeks, 15 to 17 students participate in the rotation. The students are divided into four groups of four or five people. They rotate through specific clinics and/or small hospitals in community-based settings and/or the central community hospital in each region, but not all locations in the region. Some groups of students participate in "community diagnosis" as a part of their CBME, in which they aim to provide a quantitative and qualitative description of the health of local citizens and the factors which influence their health. In this process, students collect quantitative and qualitative data concerning X hospital and/or Y clinic in different regions through windshield surveys, including observations made from a moving vehicle, interviews with local residents, and retrieval of local data in public repositories. All students also spend one week in the family medicine department of a university hospital. All settings are located in suburban or rural areas of Ibaraki Prefecture, Japan. The first and last days of the 4-week curriculum include meeting at the university hospital for orientation and reflection. During the rotation, students experience outpatient medical interviews and reflection; participate in-home visits for medical and nursing care; provide health promotion classes to citizens or elementary or junior high school students; and experience community diagnosis in various settings. The curriculum is organized by faculty members of the Department of Family Medicine at the University of School of Medicine and Health Science and is supported by physicians and other healthcare professionals, and local citizens in the community. The students are evaluated based on their performance in each setting and the final reflective report they write and submit on the final summary day.

### Procedure

**Figure 1:**
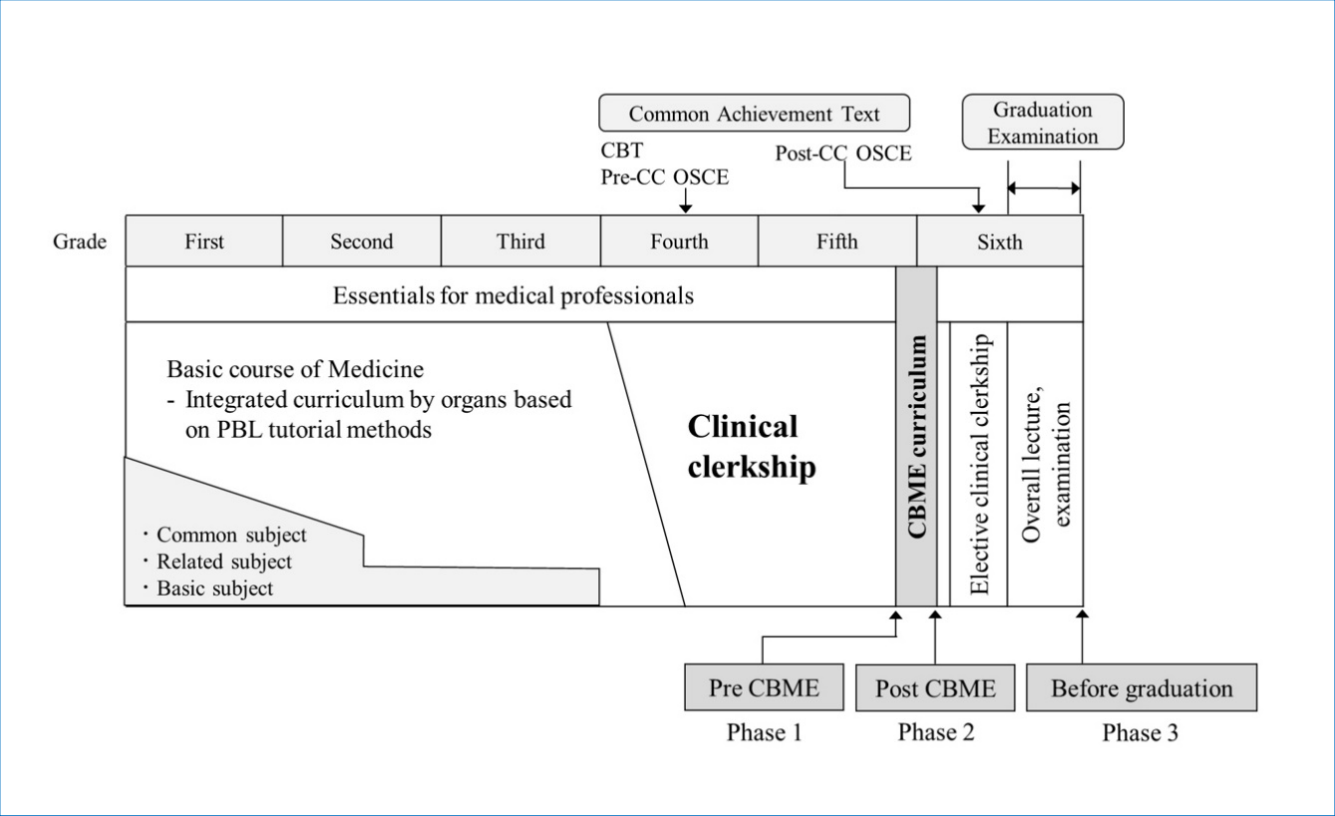
Medical education curriculum at University of Tsukuba, including a mandatory 4-week CBME clinical practice

To assess the construct validity of the developed PSIQ over time, a PSIQ survey was conducted in the pre-CBME curriculum (phase 1; before CBME on the first day of the program), post-CBME curriculum (phase 2; after CBME on the last day of the program), and delayed post-CBME curriculum (phase 3; at the time of graduation in January 2020) (Figure 1).

In phase 1, the researchers (JH and/or TM and/or AT) informed students of the aims of the study. The researchers explained that there was no detriment to not consenting, and those agreeing to participate then signed a consent form permitting the use of their data in the study. The researchers then requested that the medical students complete the PSIQ. In phase 2, as in phase 1, the students were requested to answer the PSIQ on the final day of CBME. In phase 3, all medical students answered the PSIQ on the web.

### Data analysis

First, we developed an overview of the transition of professional identity formation of each item in descriptive analysis. We also calculated Cronbach's alpha as a measure of internal reliability to illustrate intrinsic consistency.

Second, hypothesis testing for construct validity was checked by comparisons over time. We adopted repeated measures ANOVA (Analysis of Variance) by using the average of the nine items measured by PSIQ at the three-time phases. The greenhouse-Geisser correction was used to evaluate F ratios for repeated measures involving more than one degree of freedom.

Third, we hypothesized that the score for cultural awareness identity would increase between before and after the CBME curriculum. We used the paired sample t-test and calculated the standardized effect size (Cohen's d) to compare the average of the nine items measured by PSIQ before and after the CBME curriculum (phase 1-phase 2). Regarding suggested effect size values, these were 0.2 (small), 0.5 (medium), and 0.8 (large).^23^

Data analysis was conducted using SPSS ver.26, with significance set at p<0.05.

## Results

One hundred five of a total of 118 medical students (89%) who responded at the three time points were eligible for analysis. Of these 105, 33 (31.4%) were women. By activity, the number of students who trained at the university hospital, central community hospitals in each region, small community hospitals, community clinics, and who conducted community diagnosis around X community hospital and Y community clinic was 105 (100%), 35 (33.3%), 16 (15.2%), 78 (74.3%), 80 (76.2%), and 41 (39.0%), respectively (Table 1).

Internal consistency of the questionnaire as measured by Cronbach's alpha was 0.932 at phase 1, 0.936 at phase 2, and 0.939 at phase 3.

**Table 1:** Participant characteristics and location of the CBME curriculum as a percentage of the sample

Variable	Medical Student (n=105)
Gender	
Men	68.6
Women	31.4
Location (including duplications)	
University hospital	100
Community central hospital in regions	33.3
Small community hospital	15.2
Community clinics	74.3
Community diagnosis around X community hospital	76.2
Community diagnosis around Y community clinic	39.0

Table 2 summarizes the medical students' responses to the PSIQ. In Table 2, the average range of score for each item, within a possible range of 1 (low) to 7 (high), was 2.6-3.7, 3.0-4.1, and 3.8-4.7 for phases 1, 2 and 3, respectively. In Phase 1, the scores for "dealing with emergency" (2.6±1.2) and "cultural awareness" (2.9±1.2) were low. The scores for "dealing with emergency" had the lowest degree of increase (2.6±1.2, 3.0±1.2, 3.8±1.3).

**Table 2:** The mean and standard deviation of the nine items measured by PSIQ score trends in phases 1, 2 and 3

Scale	Phase 1		Phase 2		Phase 3	
	M	SD	M	SD	M	SD
Total (n=105)	29.2	8.2	33.4	8	39.0	8.2
Teamwork	3.1	1.2	3.6	1.1	4.4	1.0
Communication	3.7	1.1	4.1	1.1	4.7	1.0
Conducting assessment	3.2	1.1	3.6	1.0	4.4	1.1
Cultural awareness	2.9	1.2	3.7	1.1	4.2	1.3
Ethical awareness	3.5	1.2	4.0	1.1	4.5	1.1
Using records	3.5	1.0	3.9	1.0	4.5	1.1
Dealing with emergencies	2.6	1.2	3.0	1.2	3.8	1.3
Reflection	3.4	1.1	3.9	1.1	4.3	1.1
Teaching	3.2	1.0	3.5	1.0	4.1	1.0

PSIQ ranges from 1 (low) to 7 (high), M and SD represent mean and standard deviation, respectively. Phase 1; before CBME on the first day of the program, Phase 2; after CBME on the last day of the program, Phase 3; delayed post-CBME at the time of graduation

The scores increased progressively across the three phases. The F-test for repeated measures ANOVA of the average of the nine scores between phase 1, phase 2, and phase 3 showed a significant difference F_(__2,208)_ = 63.59, p <0.001. See Table 3.

**Table 3:** Repeated-measures ANOVA for difference in the average scores of the nine items measured by PSIQ score trends in phases 1, 2 and 3

Source	Sum of Squares	*df*	Mean Square	F	p
Conditions	63.587	2	31.794	76.173	<0.001
Error	86.816	208	0.417		

Phase 1; before CBME on the first day of the program, Phase 2; after CBME on the last day of the program, Phase 3; delayed post-CBME at the time of graduation

Moreover, there were statistically significant differences of all average of the nine items measured by PSIQ before and after the CBME curriculum (phase 1-phase 2). The effect sizes of each item in phases 1-2 were 0.30-0.67. Only the effect size of cultural awareness (0.68) was medium between phase 1 and phase 2; the effect sizes of the others between these phases were less than 0.5 (before and after CBME) (Table 4).

## Discussion

In this study, we validated the PSIQ for medical students in clinical practice, which included CBME, and demonstrated that PSIQ scores increased over time.

First, the PSIQ has simple but well-defined content. The validity of the PSIQ was demonstrated by the findings in the same individuals at the three times phases. The original paper assessed multifaceted professional identity among medical students in the United Kingdom,1 and the measure has been associated with pre-and post-test behaviors in non-physicians. For example, a study of pharmacists in the U.S. showed that pharmacy work experience prior to matriculation into pharmacy school created a stronger sense of professional identity.^20^ Our present study, in which PSIQ was partially validated during clinical practice in Japan, suggests that it could be used not only across professions but also across countries and cultures.

**Table 4:** Paired sample t-test and standardized effect size (Cohen's d) in phases 1 and 2

Scale	*t*_(104)_	p	95%CI		Cohen's d
			LL	UL	
Teamwork	3.72	<.001	0.20	0.67	0.42
Communication	4.15	<.001	0.23	0.65	0.36
Conducting assessment	4.28	<.001	0.23	0.63	0.36
Cultural awareness	6.46	<.001	0.51	0.96	0.67
Ethical awareness	4.35	<.001	0.29	0.78	0.42
Using records	3.68	<.001	0.18	0.60	0.40
Dealing with emergencies	4.00	<.001	0.21	0.61	0.33
Reflection	4.69	<.001	0.29	0.72	0.46
Teaching	3.21	0.002	0.12	0.49	0.30

LL and UL represent the lower- limit and upper limit of a confidence interval, respectively

Second, professional identity as measured by the PSIQ in this study showed a certain internal consistency, as measured by Cronbach's alpha during the same period of clinical practice. As would be expected, PSIQ scores increased with time. Although some aspects of personal identity are relatively stable throughout life, professional identity is reported to change more dynamically,^24^ and is implicit, unstable, and indeterminate. In a previous study, implicit change in professional identity was manifested using the PSIQ, and used as a criterion when selecting interviewees.^25^ This use suggests that the professional identity of individuals can be evaluated by obtaining feedback on their professional identity during clarification of professional identity formation, and that the results can then be used to evaluate individual positions within the same grade group.

Third, as a CBME-related consideration, the CBME curriculum may stimulate a professional identity that differs from that for hospital practice. Previous findings have also shown that the CBME curriculum provides a rich contextual environment in which to promote the development of meaningful relationships.^26^ In particular, CBME requires the learning of social behavior, anthropology, illness, culture, and communication, which are all considered community-orientation elements.^27^ Given that our present study assessed multiple professional identities relative to each other, the role identity of "cultural awareness" might have shown the greatest increase among the identities during CBME. Thus, these findings may reflect the benefits of CBME to the future abilities of students in clinical practice, and the PSIQ may provide evidence that can be used for curriculum evaluation.

Several limitations of our study warrant mention. The study was conducted as a single-university, single-year longitudinal questionnaire study in Japan and the timings of phases 1, 2 and 3 differed among the medical student participants. The findings should therefore be analyzed with caution. Additionally, depending on the timing of the CBME curriculum, the period between phases 1-2 and phases 2-3 among participants ranged from 8 to up to 15 months, and PSIQ scores may be affected by differences in regional learning experiences among students during CBME. Accordingly, it is possible that student responses might have varied depending on when and where they actually took the CBME curriculum. Despite these limitations, the identification of professional identity formation with multiple components during clinical practice, including CBME, may provide an opportunity for meaningful feedback on the readiness to become a doctor for both medical students and faculty. Given that physicians are required to handle uncertain and complex events, the strategic construction of a CBME curriculum that enhances the professional identity of cultural awareness in clinical practice may have a beneficial impact on the professional identity of future physicians. Further studies are warranted to explore how medical students develop professional identity during clinical practice, including CBME, and on what factors impact medical students and faculty in understanding the trajectory of professional identity formation.

## Conclusions

We validated the PSIQ for medical students in clinical practice, and demonstrated that PSIQ scores increased over time. This tool can serve as feedback on professional identity formation to students and faculty in clinical practice. Reflection on professional identity formation using the PSIQ may provide an opportunity for meaningful feedback on the readiness to become a doctor. Further use of the PSIQ at multiple universities and in various types of clinical practice will allow assessment of its robustness as a tool, and its impact on students and faculty.

### Acknowledgments

We thank the faculty staff Shoji Yokoya, Hisashi Yoshimoto, Naoto Sakamoto, Yu Yamamoto, Yoshihiro Kataoka, Shoichi Masumoto, Haruka Kuno, Takashi Inaba, Shuhei Hamada, Shogo Kawada, and Sayaka Nin of the Department of Primary Care and Medical Education, Faculty of Medicine, University of Tsukuba. Additionally, we thank the healthcare staff who taught the medical students at the CBME curriculum sites in clinical practice. The education/research fund of Department of Primary Care and Medical Education, Faculty of Medicine, University of Tsukuba.

### Conflicts of Interest

The authors declare that they have no conflict of interest.

## References

[1] Crossley J, Vivekananda-Schmidt P (2009). The development and evaluation of a Professional Self Identity Questionnaire to measure evolving professional self-identity in health and social care students.. Med Teach.

[2] Adams K, Hean S, Sturgis P, Clark JM (2006). Investigating the factors influencing professional identity of first-year health and social care students.. Learn Health Soc Care.

[3] Ashby SE, Adler J, Herbert L (2016). An exploratory international study into occupational therapy students' perceptions of professional identity.. Aust Occup Ther J.

[4] El Chakhtoura NG, Bonomo RA, Jump RLP (2017). Influence of aging and environment on presentation of infection in older adults.. Infect Dis Clin North Am.

[5] Cruess RL, Cruess SR, Boudreau JD, Snell L, Steinert Y (2015). A schematic representation of the professional identity formation and socialization of medical students and residents: a guide for medical educators.. Acad Med.

[6] Weaver R, Peters K, Koch J, Wilson I (2011). 'Part of the team': professional identity and social exclusivity in medical students.. Med Educ.

[7] Goldie J (2012). The formation of professional identity in medical students: considerations for educators.. Med Teach.

[8] Wilson I, Cowin LS, Johnson M, Young H (2013). Professional identity in medical students: pedagogical challenges to medical education.. Teach Learn Med.

[9] Siebert DC, Siebert CF (2005). The caregiver role identity scale: a validation study. Research on Social Work Practice.

[10] Jarvis-Selinger S, Pratt DD, Regehr G (2012). Competency is not enough: integrating identity formation into the medical education discourse.. Acad Med.

[11] Apker J, Eggly S (2004). Communicating professional identity in medical socialization: considering the ideological discourse of morning report.. Qual Health Res.

[12] Haidet P, Hatem DS, Fecile ML, Stein HF, Haley HL, Kimmel B, Mossbarger DL, Inui TS (2008). The role of relationships in the professional formation of physicians: case report and illustration of an elicitation technique.. Patient Educ Couns.

[13] Mennin S, Petroni-Mennin R (2006). Community-based medical education.. Clin Teach.

[14] Haruta J, Yamamoto Y (2020). Realist approach to evaluating an interprofessional education program for medical students in clinical practice at a community hospital.. Med Teach.

[15] Ahmad A, Bahri Yusoff MS, Zahiruddin Wan Mohammad WM, Mat Nor MZ (2018). Nurturing professional identity through a community based education program: medical students experience.. J Taibah Univ Med Sci.

[16] Hunt JB, Bonham C, Jones L (2011). Understanding the goals of service learning and community-based medical education: a systematic review.. Acad Med.

[17] Mariam DH, Sagay AS, Arubaku W, Bailey RJ, Baingana RK, Burani A, Couper ID, Deery CB, de Villiers M, Matsika A, Mogodi MS, Mteta KA, Talib ZM (2014). Community-based education programs in Africa: faculty experience within the Medical Education Partnership Initiative (MEPI) network.. Acad Med.

[18] Henderson M, Upham S, King D, Dick ML, van Driel M (2018). Medical students, early general practice placements and positive supervisor experiences.. Educ Prim Care.

[19] Worthington M, Salamonson Y, Weaver R, Cleary M (2013). Predictive validity of the Macleod Clark Professional Identity Scale for undergraduate nursing students.. Nurse Educ Today.

[20] Bloom TJ, Smith JD, Rich W (2017). Impact of pre-pharmacy work experience on development of professional identity in student pharmacists.. Am J Pharm Educ.

[21] Suzuki Y, Gibbs T, Fujisaki K (2008). Medical education in Japan: a challenge to the healthcare system.. Med Teach.

[22] Nara N, Suzuki T, Tohda S (2011). The current medical education system in the world.. J Med Dent Sci.

[23] Leppink J, O'Sullivan P, Winston K (2016). Effect size - large, medium, and small.. Perspect Med Educ.

[24] Vignoles VL, Schwartz SJ, Luyckx K. Introduction: toward an integrative view of identity. In: Schwartz SJ, Luyckx K, Vignoles VL, editors. Handbook of identity theory and research. 2011.

[25] Vivekananda-Schmidt P, Crossley J, Murdoch-Eaton D (2015). A model of professional self-identity formation in student doctors and dentists: a mixed method study.. BMC Med Educ.

[26] Kelly L, Walters L, Rosenthal D (2014). Community-based medical education: is success a result of meaningful personal learning experiences?. Educ Health (Abingdon).

[27] Claramita M, Setiawati EP, Kristina TN, Emilia O, van der Vleuten C (2019). Community-based educational design for undergraduate medical education: a grounded theory study.. BMC Med Educ.

